# Transarticular Screw Fixation in the Treatment of Severe C1–C2 Dislocation: A Case Series Report

**DOI:** 10.1111/os.12792

**Published:** 2020-11-13

**Authors:** Hoang Gia Du, Vu Xuan Phuoc, Nguyen Duc Hoang, Tran Trung Dung, Nguyen Van Trung

**Affiliations:** ^1^ Hanoi Medical University Hanoi Vietnam; ^2^ Department of Orthopedics and Spine Bachmai University Hospital Hanoi Vietnam; ^3^ Saint Paul University Hospital Hanoi Vietnam; ^4^ Orthopaedic Division, Faculty of Surgery Hanoi Medical University Hospital Hanoi Vietnam

**Keywords:** Atlantoaxial dislocation, Bone autoplasty, C1–C2 transarticular screw fixation, Halo traction

## Abstract

**Background:**

To aim of the present paper was to evaluate the results of halo traction and transarticular screw fixation combined with bone autoplasty in patients with severe atlantoaxial dislocation.

**Case presentation:**

This is a retrospective study of severe cases of atlantoaxial dislocation in nine patients (six men and three women) treated with preoperative halo traction and posterior C1–C2 transarticular screw fixation combined with bone autoplasty from June 2006 to June 2011 at the Saint Paul Hospital (Hanoi). The mean age of patients was 37.48 ± 13.753 years (range, 26–50 years). The possibility of fixing dislocation using a halo apparatus was investigated through a series of preoperative halo corrections performed within a span of 1–2 weeks. For transarticular screw fixation, two transarticular screws were used that were positioned according to the Magerl technique. For bone autoplasty, an iliac crest bone graft approximately 3 × 2 cm in size was used. The postoperative assessment of clinical improvement was performed using the neck disability index (NDI), the American Spinal Injury Association (ASIA) impairment scale, and the visual analog scale (VAS) measurement instruments, through the gradation of atlantoaxial dislocation, and *via* the clivoaxial angle(CAA) index and the space available for cord (SAC) index after 6 months. The image diagnosis demonstrates that all the cases of atlantoaxial dislocations are unstable and correspond to the Fielding and Hawkins type III dislocation. Eight patients underwent complete reduction using the halo fixation device. In one patient, the C1–C2 displacement was manually reduced during surgery. CT scanning revealed that the accuracy of screw placement was 94.4%. The bone fusion rate was 100% after 6 months. Based on the ASIA impairment scale, the preoperative examination of patients revealed grade C injuries in seven patients and grade D injuries in two patients. After surgery, all patients had grade D injuries. Six months after surgery, four patients had moderate self‐reported neck disability (30%–48%) and five patients reported mild disability (10%–28%); that is, the patient perception of the neck problem improved. In the postoperative phase, all patients showed an improvement in VAS pain scores and the SAC score returned to the normal range in all patients. The CAA returned to normal in only seven patients; in the other two patients, the CAA returned to a value that was close to normal (145° and 149°).

**Conclusion:**

Through halo traction combined with transarticular screw fixation and bone autoplasty, noticeable postoperative improvements were attained based on the clinical scores for NDI, ASIA, and VAS, as well as SAC and CAA.

## Graphical abstract

The purpose of the research was to evaluate the results of halo traction and transarticular screw fixation combined with bone autoplasty in severe atlantoaxial dislocation patients. Through halo traction combined with transarticular screw fixation and bone autoplasty, the noticeable postoperative improvements were attained based on the clinical scores for NDI, ASIA, and VAS, as well as SAC and CAA.

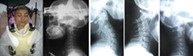

## Introduction

Injuries to the cervical spine are serious and are distinguished by diversity, high risk of severe neurological complications, and mortalityCervical spinal cord injuries make up 50% to 75% of all spinal cord injuries^1^, ^2^, ^5^. In turn, different injuries to the craniovertebral junction account for up to 20% of all cervical injuries^6^. The C1–C2 complex is stabilized by three major stabilizers, the odontoid process, the cruciate ligament, and the post‐primary ligamentous apparatus (alar and joint capsular ligaments)^7^. Damage to these structures due to trauma (e.g. odontoid fracture or tears of the transverse ligament of the atlas) or disease can cause C1–C2 instability. A further displacement of the atlas is known as an atlantoaxial dislocation (AAD). Traumatic injuries are the most common cause of AAD with a displacement of the odontoid process, accompanied by spinal stenosis and spinal cord compression^8^.

There are five types of dislocation depending on the vector: anterior, posterior, lateral, rotational, and vertical^9^, ^10^, ^11^, ^12^, ^13^, ^14^. One type may occur in conjunction with other^15^, ^16^. Anterior dislocations, in turn, are divided into transdental and transligamentous dislocations^17^. Transdental dislocations are associated with an odontoid fracture and the odontoid process moves forward with the anterior arch of the atlas. The posterior upper angle of the broken process is the underlying compression factor. Between this angle and the posterior arch of the atlas, a critical section of the spinal canal is located where the spinal cord and its great vessels are squeezed^17^. The transligamentous dislocation is associated with tears of the transverse ligament of the atlas, which normally prevents excessive displacement of the atlas. In this case, the compression substrate is the odontoid process on which the dural sac is flattened and roughly squeezed along with the spinal cord^17^. The traumatic posterior displacement of the atlas occurs only in conjunction with the posterior displacement of the odontoid process, which can roughly compress the spinal cord^17^.

Regardless of the cause of the spinal canal stenosis at the level of the craniovertebral junction, the range of surgical methods used for the treatment is diverse^18^. Surgical interventions aim to prevent neurological complications, restore the anatomical relationship of the spine, and fix the damaged segments. One of the key factors influencing the choice of treatment is the dislocation stability.^18^

The atlantoaxial instability is marked by a change in the distance between the anterior arch of the atlas (C1) and the dens of the axis (C2). CT scanning is an effective way to diagnose such displacements^19^.

There three main types of atlantoaxial instability are distinguished: flexion–extension, distraction, and rotatory^12^, ^13^, ^19^. They can occur as isolated or, more often, in various complex combinations. Instability is associated with damage to the structures of the craniocervical junction. The transverse ligament is a key component here, the integrity or damage of which defines the degree of atlantoaxial displacement and instability. If the distance between the anterior portion of the ring of C1 and the odontoid process (or dens) of C2 exceeds 7 mm, then a compression of the spinal cord occurs^13^. Compression of the ventral spinal cord is the most dangerous complication of atlantoaxial dislocations (ADD). The vertical displacement of the atlas occurs due to the C1–C2 separation. The tip of the odontoid process is normally located at the level of the anterior arc of the atlant or even more caudally^12^. The main cause of vertical instability is rupture of pterygoid ligaments and tectorial membrane^12^, ^13^. Hence, vertical instability is a manifestation of ruptures that occur at the atlanto‐occipital joint.

The traumatic rotatory displacement of the atlas can vary from insignificant to virtually complete dislocation. In the open mouth, radiographic images of the rotary displacement show an eccentric location of the dens positioned between the lateral masses, and the atlantoaxial height is asymmetrically changed. A change in the distance between the anterior part of the ring and the axis is an important indicator of stability^18^. Fielding and Howkins^19^ distinguish four types of atlantoaxial rotatory subluxation. Type I is a simple rotatory displacement with an intact transverse ligament; the dens is considered a center of rotation. These rotatory subluxations are permanent injuries. Type II is an anterior displacement of C1 on C2 between 3 and 5 mm, with one lateral mass that ensures axial rotation and an inferior transverse ligament injury. This type of damage suggests the possibility of intermittent subluxation. Type III is an anterior displacement greater than 5 mm. The lateral masses are either clearly subluxated or damaged. This fixes the C1–C2 dislocation, destabilizing the atlantoaxial articulation. Type IV is rare and manifests as a posterior displacement of C1 on C2. Such an injury usually occurs in conjunction with a broken or defective process.

The advanced neurological deficit along with a high risk of vital disorders require prompt elimination of vertebro‐medullary conflict with subsequent reliable spine stabilization. For traumatic neural compression of the upper cervical spine, craniovertebral junction repair is preferred. This may be achieved through, for instance, halo correction, skeletal traction, or manual or instrumental intraoperative repair of vertebrae^8^, ^20^.

The following items are used to stabilize the upper cervical vertebrae: suture material and wire, bone grafts, and metal and plastic accessories^21^. In recent years, stabilization of the C1–C2 complex has been carried out mostly with screws^22^, ^23^. In many cases, Magerl's technique, applying C1–C2 transarticular screw fixation with posterior wiring^24^, and its modifications remain the method of choice for reliable spine stabilization in AAD^2^, ^3^, ^4^, ^5^, ^25^, ^26^, ^27^, ^28^, ^29^. It enables prompt fixation of blocked segments^30^, ^31^, ^32^ and a level of formation higher than after dorsal spinal fusion with wiring to hold bone grafts^33^, ^34^. This article examines cases of unstable AAD treatment based on preoperative instrumental correction with the halo apparatus, posterior percutaneous screw fixation of the C1–C2 complex, and bone autoplasty. This method of treatment was assumed effective against the neurological deficit that occurs in unstable AAD accompanied by spinal stenosis and spinal cord compression.

Note that this pathology is rare and, for this reason, does not allow for large‐scale studies. Nevertheless, severe AAD cases pose a threat to the lives of patients, which demands the identification of the most effective treatment. For this reason, the main aim of this study is to evaluate the outcomes after halo traction and transarticular screw fixation combined with autoplasty in severe atlantoaxial dislocation patients. To accomplish this goal, the following objectives were set:

1. Conduct preoperative clinical diagnosis and imaging tests to categorize dislocation and identify indications for transarticular screw fixation.
2. Perform preoperative instrumental correction with the use of the halo traction and subsequent transarticular screw fixation.
3. Six months after the surgical intervention, evaluate bone fusion rates, clinical improvements in neck disability index (NDI), American Spinal Injury Association (ASIA) impairment scale, and visual analogue scale (VAS), as well as imaging findings.

## Case Reports

### *Materials and Methods*

*Inclusion criteria*

1. Radiographically visible damage to elements ensuring C1–C2 stability that is associated with injury.
2. ADD, diagnosed as unstable.
3. Transverse ligament disruption.
4. Pronounced neurological deficit (incomplete motor function on ASIA scale).

*Exclusion criteria*

1. Rheumatoid changes of the craniovertebral region.
2. Bone defects and fractures in the skull.
3. The presence of contraindications for the application of the halo apparatus: restricted movement due to the weight of equipment, injury to soft tissues, and bones at pin sites and under the halo vest.

#### *Subjects*

This is a retrospective study of severe ADD cases in nine patients (six men and three women) treated with preoperative halo traction and posterior C1–C2 transarticular screw fixation combined with bone autoplasty between June 2006 and June 2011 at the Saint Paul Hospital (Hanoi). The mean age of patients was 37.48 ± 13.753 years (range, 26–50 years).

All patient investigations conformed to the principles outlined in the Declaration of Helsinki and were performed with permission from the Ethics Committee of Saint Paul Hospital (Hanoi). All patients provided written informed consent.

#### *Clinical Examination, and X‐ray and Radiographic Diagnostics*

All patients were examined for clinical symptoms and their neurological status was assessed using NDI (neck dysfunction), ASIA (neurological deterioration), and VAS (pain intensity) instruments. The musculoskeletal system was also investigated.

The ASIA impairment scale is the gold standard for assessing spinal cord injuries^35^. The exam is based on neurological responses, touch and pinprick sensations tested in each dermatome, and the strength of the muscles that control key motions on both sides of the body. Muscle strength is scored on a scale of 0 to 5, and sensation is graded on a scale of 0 to 2, where “0” is no sensation, “1” is altered or decreased sensation, and “2” is full sensation. Each side of the body is graded independently. The completeness or incompleteness of the injury is measured using the ASIA impairment scale:

Grade A: Complete injury; no motor or sensory function is preserved in the sacral segments S4 or S5.

Grade B: Sensory incomplete; sensory but not motor function is preserved below the level of injury, including the sacral segments.

Grade C: Motor incomplete (motor function is preserved below the level of injury, and more than half of the muscles tested below the level of injury have a muscle grade less than 3 in muscle strength scores).

Grade D: Motor incomplete (motor function is preserved below the level of injury and at least half of the key muscles below the neurological level have a muscle grade of 3 or more in muscle strength scores).

Grade E: Normal. No motor or sensory deficits, but deficits existed in the past.

The NDI is a 10‐item self‐report questionnaire covering pain intensity, personal care, lifting, reading, headache, concentration, work status, driving, sleeping, and recreation. Each item is scored on a scale from 0 (no disability) to 5 (complete disability). The total score was multiplied by 2 to produce a percentage score. The lower the score, the lesser the self‐rated disability^36^, ^37^.

The pain VAS is a unidimensional self‐reported measure of pain intensity. The VAS is a 10 point scale, where 0 points indicates no pain and 10 points indicates very severe pain.

The preoperative radiographic examination included plain radiography, CT imaging, and MRI of the cervical spine.

All patients underwent conventional radiography (lateral and medial projections, with open mouth, bending) and 64‐slice CT scans. A displacement of the lateral masses exceeding 7 mm or an increase in the atlantodental interval greater than 3 mm suggested disruption of the transverse ligament of the atlas.

CT scanning with axial, coronal, and sagittal thin‐cut (1 mm) image reconstruction was performed (i) to determine the anatomy of the cervical spine; (ii) to delineate the position of openings in the transverse processes through which the vertebral artery passes: and (iii) to measure the necessary length of screws to be implanted.

MRI was used to diagnose the spinal cord injury and the injury to the transverse ligament.

C1–C2 mobility was assessed using dynamic X‐ray and the spinal cord injury mobility was assessed using the clivoaxial angle (CAA) index and the space available for cord (SAC) index.

**Fig. 1:**
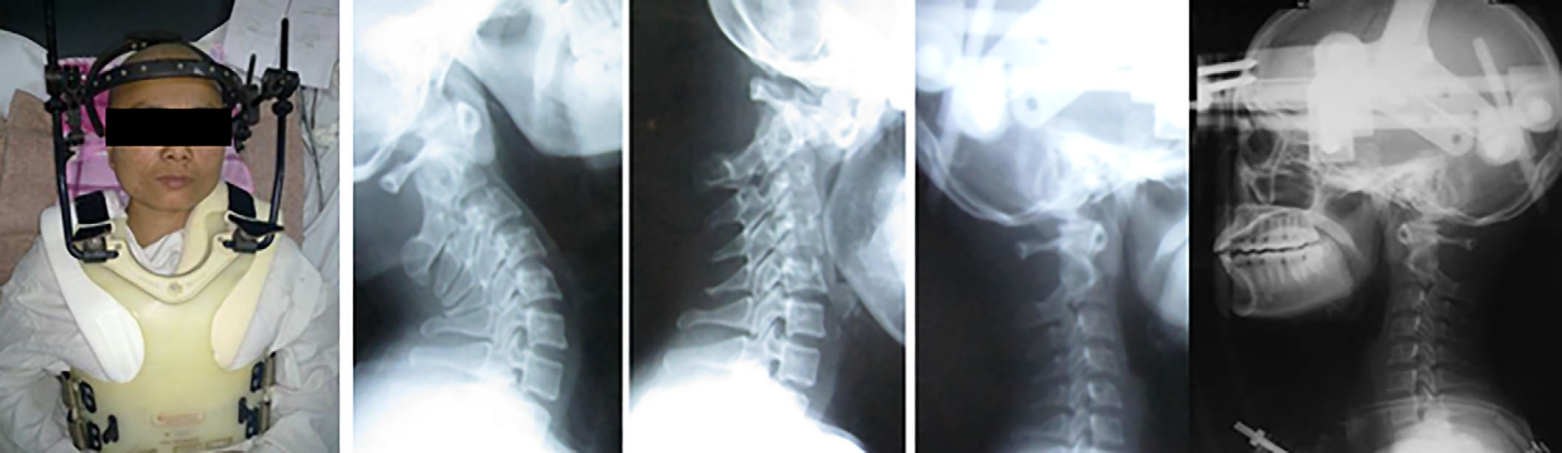
Normal clivoaxial angle (CAA).

The CAA is the angle between the clivus, a bony part of the base of the skull, and the spine. It is used to aid in the diagnosis of craniocervical instability. It is sensitive to horizontal instability and characterizes the relationships among the skull, the brainstem, and the odontoid process, including abnormalities due, for example, to a compromised transverse ligament. The average CAA in the healthy or the nonsymptomatic population is estimated to be approximately 150°; in the neutral position, the CAA varies from 150° to 165° (Fig. 1). A consensus statement formed at the second International CSF Dynamics Symposium of the Chiari and Syringomyelia Foundation in 2013 proposed that a CAA lower than 135° was potentially pathological.

The SAC is calculated by subtracting the sagittal diameter of the spinal cord from the sagittal diameter of the spinal canal. The sagittal spinal cord diameter is measured from the posterior wall of the vertebral body (halfway down) to the laminar line. The sagittal diameter of the spinal cord is usually measured from an MRI. SAC measurements of >18 mm = normal; 15 to 17 mm = possible abnormality; <14 mm = spinal cord compression, with a threat of developing cervical myelopathy.

#### *Halo Correction*

**Fig. 2:**
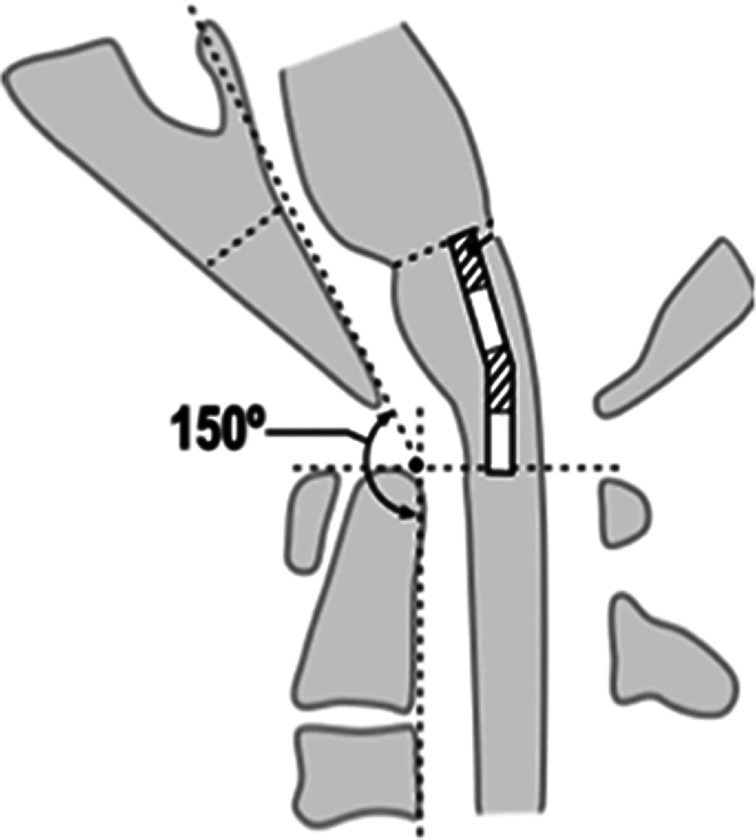
Halo traction and correction after the X‐ray exam and a case study.

All patients with severe ADD lasting for 1 to 2 weeks underwent halo traction (Fig. 2). The halo frame was adjusted after an X‐ray exam. During and after halo traction, patients were checked for status changes. The degree of atlantoaxial instability, neurological outcomes, ASIA scores, and complications were assessed.

#### *Transarticular Screw Fixation*

This technique combines the placement of two transarticular screws for the fixation of C1–C2 complex with bone grafting.

**Fig. 3:**
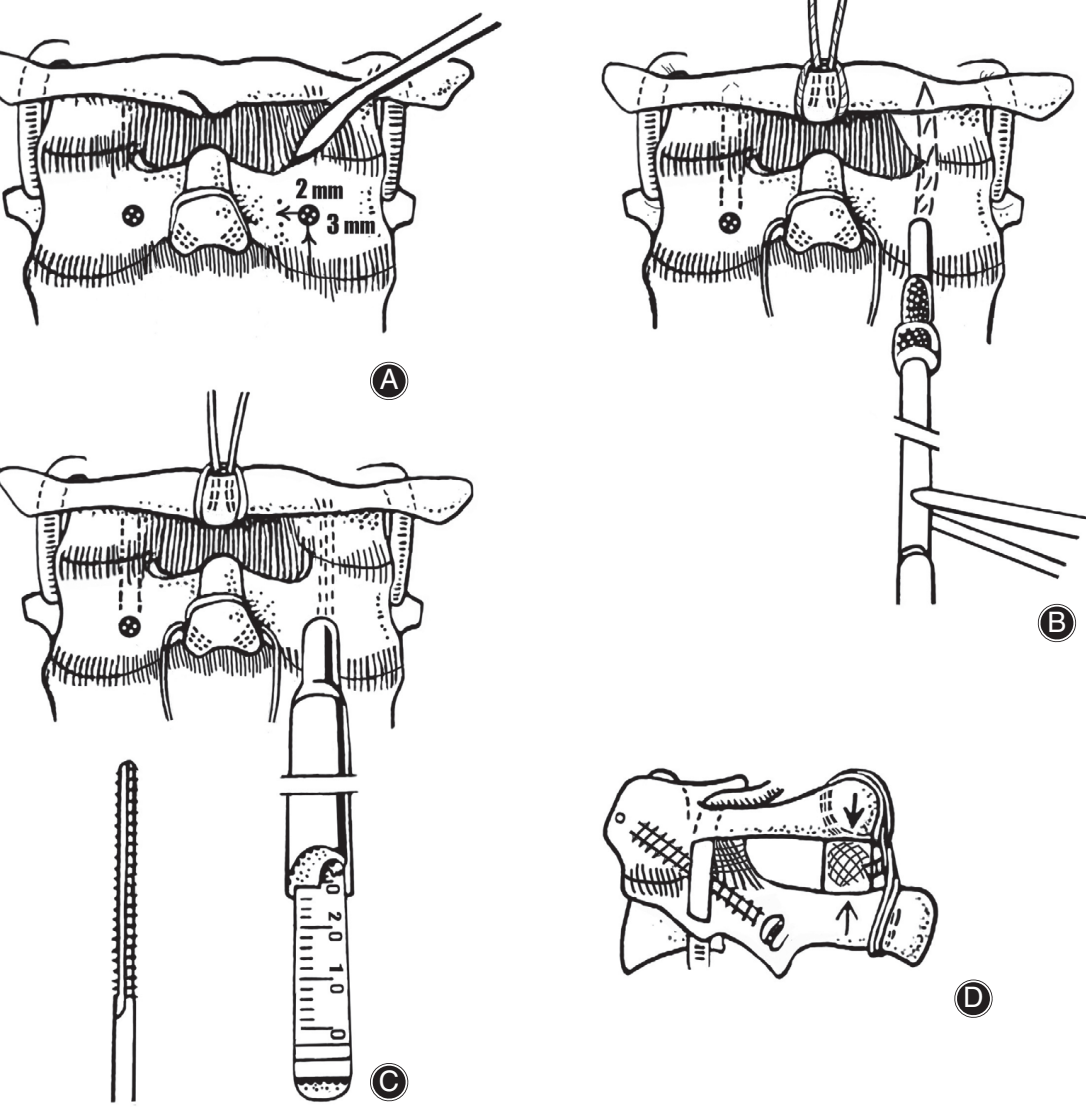
Transarticular C1–C2 screw fixation procedure: the placement of two transarticular screws with bone grafting. (А) Detachment of the upper surface of the arch and the isthmus of C2 with a small dissector; subperiosteal detachment of the posterior capsule of the atlantoaxial joint. The starting point for the drill is located close to the lower edge of the caudal articular process of C2. (B) A drill passing into the lateral mass of the atlas through the isthmus near the medial dorsal layer of the C2 articular process. (С) Screw length measurement and tapping with a 3.5‐mm tap. (D) Dorsal C1–C2 fusion following the bilateral screw fixation; spongy‐cortical bone grafting is conducted in conjunction with the dorsal cerclage.

1. Tracheal intubation was performed through the nose and a nasogastric tube tube was inserted afterwards. For the C1–C2 transarticular screw fixation, patients were positioned prone on a soft round pillow with the halo traction maintained. Fixation of the halo ring was performed using an adapter attached to the Mayfield head holder with the neck in neutral position.
2. In coordination with the anesthetist, the surgeon stabilized the patient's head. The patient was carefully turned prone onto the operating table and the pillow was placed under his chest. The halo ring was affixed to the operating table using a specific holding device, while the cervical spine was maintained in a neutral position. Soft pillows were placed under all bony prominences. The patient's hands were secured with a folded sheet that was tucked under the body of the patient.
3. The relative position of C1 and C2 was fluoroscopically evaluated with the radiograph centered at the segment C1–C2. The lateral X‐ray projections were confirmed to be not oblique.
4. A small dissector was used to detach the upper surface of the arch and the isthmus of C2. The posterior capsule of the atlantoaxial joint was exposed to subperiosteal detachment. The C2 entry point was identified by first locating the medial edge of the C2–C3 facet joint. The C2 entry site is just lateral and rostral to this point, and may be estimated by visualizing the course of the medial pars (approximately 3 mm up and 2 mm out) (Fig. 3A). A drill was passed through the isthmus near the medial dorsal layer of the C2 articular process.
5. The placement of transarticular screws was similar in technical detail to the technique described by Magerl *et al*. in 1979^24^. A 2‐mm drill through with a tip pointing was used to drill a bicortical pilot hole in a stab incision lateral to the C1 spinous process directed 15° medially, with the superior angle visualized by fluoroscopy. The drill was directed down the C2 pedicle and across the C1–C2 joint, aimed at the anterior tubercle of C1. The tip of the drill was advanced to a point 4 mm short of the anterior C1 tubercle, attaining purchase of the anterior cortex of C1 (Fig. 3B). Horizontal drilling was avoided because the vertebral artery at the level of C2 was directed up and anterior to the C1–C2 joint and could be damaged. Furthermore, the screw may ventrally extend beyond C2 from the ventral side and not enter the atlas.
6. The necessary screw length was measured directly from the drill and a thread was cut into a hole with a 3.5‐mm cortical tap (Fig. 3C). After tapping, a fully threaded 3.5‐mm diameter cortical screw was used. Screws were typically 34 to 44 mm in length. The technique was repeated on the contralateral side.
7. Harvested from the posterior iliac crest, the iliac crest bone graft was approximately 3 × 2 cm. The lamina of the C2 vertebra and C1 arch were decorticated before application of the bone graft with a high‐speed burr. Bone graft was placed and secured between the posterior arch of C1 and the spinous process of the C2 vertebra with a thread using the Gallie technique (Fig. 3D).
8. The halo traction was removed postoperatively and all patients were kept in a rigid collar for 6 weeks.

#### *Postoperative Assessment of Clinical Improvement*

Six months after treatment, postoperative assessment of clinical improvement and imaging diagnostic criteria was carried out using the NDI, ASIA, and VAS (visual analog scale), as well as the CAA index and the SAC index.

#### *Statistical Analysis*

Data processing was performed in Excel 2016 (Microsoft, USA). Means and standard deviations were calculated.

## Results

### *Diagnostic Imaging*

The image diagnosis demonstrates that all the cases of atlantoaxial dislocations are unstable and correspond to the Fielding and Hawkins type III dislocation^19^. This type of dislocation is characterized by a rotatory and anterior displacement greater than 5 mm with both lateral atlantoaxial joints anteriorly subluxed; the transverse ligament and facet capsules are injured.

### *Halo Correction*

**Fig. 4:**
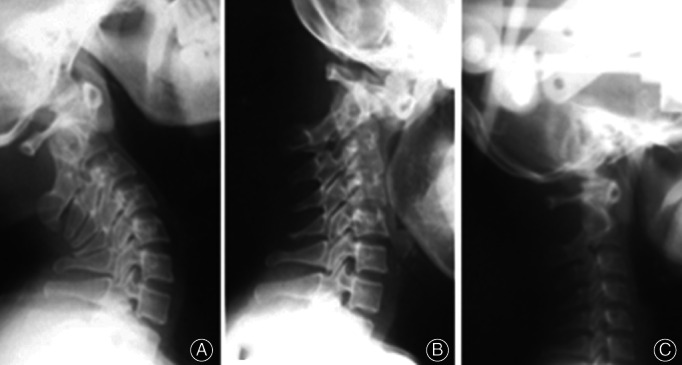
C1–C2 dislocation on X‐ray before (A, B) and after (C) halo traction.

First, patients were examined for the possibility of fixing dislocation by using a halo apparatus. All dislocations were classified as halo‐corrected or halo‐uncorrectable^20^. Generally, reposition is possible in acute traumatic dislocations. However, halo correction is usually ineffective in chronic injuries or rheumatoid pannus due to rigid chronic vertebral displacement. In these situations, decompression is achieved by compressing substrate resection *via* a ventral surgical approach^21^. The craniovertebral junction was successfully restored (halo‐corrected dislocation) in eight patients (Fig. 4). There was one case of incomplete reposition in which C1 displacement remained anterior to C2 (Fielding and Hawkins type I dislocation) and was manually reduced during surgery.

### *Postoperative Outcomes*

**Fig. 5:**
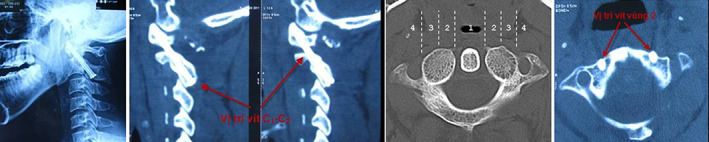
C1–C2 screw position on vertical (A, B) and horizontal (C) CT scanner film.

CT scanning revealed that the accuracy of screw placement was 94.4%. (Fig. 5). At the 6‐month follow‐up, the bone fusion rate was 100%.

Postoperative outcomes 6 months after the intervention are presented in Table 1.

**TABLE 1:** Postoperative assessment of clinical improvement and imaging diagnostic criteria

Case number	Age	Sex	Causes of injury	ASIA		NDI (%)		VAS		SAC (mm)		CAA (degree)	
				Preoperative	Postoperative	Preoperative	Postoperative	Preoperative	Postoperative	Preoperative	Postoperative	Preoperative	Postoperative
1	35	F	Motobike	C	D	64	35	7	3	3	22	90	160
2	50	M	Fall	C	D	40	25	5	2	6	20	115	145
3	41	F	Fall	C	D	45	22	6	1	5	16	120	152
4	40	M	Motobike	C	D	35	18	5	2	5	16	121	160
5	37	M	Fall	C	D	60	40	7	3	5	15	95	155
6	38	M	Fall	D	D	35	17	4	1	7	22	111	158
7	31	M	Motobike	C	D	55	35	6	2	3	19	97	154
8	39	M	Motobike	C	D	34	25	5	1	7	20	118	149
9	26	F	Fall	D	D	40	30	5	1	5	18	106	150

ASIA, American Spinal Injury Association impairment scale; CAA, clivoaxial angle; NDI, neck disability index; SAC, space available for cord index; VAS, visual analog scale.

The ASIA impairment scale is used to classify the severity of spinal cord injuries^35^. Prior to the operation, the ASIA exam revealed:

1. Seven patients with grade C injuries. Motor function was preserved below the neurological level, and more than half of key muscles below the neurological level had a muscle grade below 3 (active movement against gravity throughout the full range of motion).
2. Two patients with grade D injuries. Motor function was preserved below the neurological level, and at least half of the key muscles below the neurological level had a muscle grade of 3 or higher (active movement against gravity throughout the full range of motion).

Postoperatively, all patients ended up with grade D injuries, which indicates improvement in the ASIA score in most patients.

The mean preoperative NDI finding was 46% ± 12.1% (range, 34%–64%), with three patients having severe disability (range, 50%–64%) and six patients reporting moderate disability (range, 34%–48%). Six months after treatment, the postoperative mean was 27.13% ± 8.5% (range, 17%–40%), by contrast, with four cases of moderate disability (30%–40%) and five cases of mild disability (17%–29%). In other words, patients reported a fair improvement in self‐assessed functional status (*P* < 0.0001).

As can be seen from Table 1, the mean VAS showed a general improvement, from 5.6 ± 1.1 (range, 4–7) to 1.9 ± 0.8 (range, 1–3). No patient had SAC measurement of >14 mm before surgery (mean, 5.13 ± 1.56 mm; range, 3 to 7 mm). After posterior transarticular screw fixation, the sagittal diameter of the spinal cord returned to normal (mean, 18.5 ± 3.5 mm; range, 15–22 mm), indicating a fair improvement (*P* < 0.0001). All patients had abnormal CAA before surgery and this predisposes the individual to biomechanical neuraxial stress, which is manifested by myelopathy, bulbar symptoms, and neck pain or headache. The mean CAA was 108.36 ± 12.44°, with the range from 90° to 121°. After the intervention, seven patients had their CAA return to normal and the remaining two patients achieved CAA close to normal (145° and 149°). The mean CAA was 154.13 ± 5.33°, with the range from 145° to 160°, *P* < 0.0001.

### *Clinical Case Number 5*

Patient A, a 37‐year‐old man, was admitted to the spine surgery unit (SSU) on 23 March 2009, 3 months after falling from a ladder during construction and repair work. Immediately after the accident, he underwent outpatient treatment, but the neck pain persisted. On admission to the SSU, he complained of restricted movement in the cervical spine, along with severe pain in the neck when turning the head, and muscle weakness in the hands. Two and a half months after the injury a CT scan of the cervical spine revealed rupture of the transverse ligament. The patient was kept in a Philadelphia collar until the scheduled surgical treatment.

**Fig. 6:**
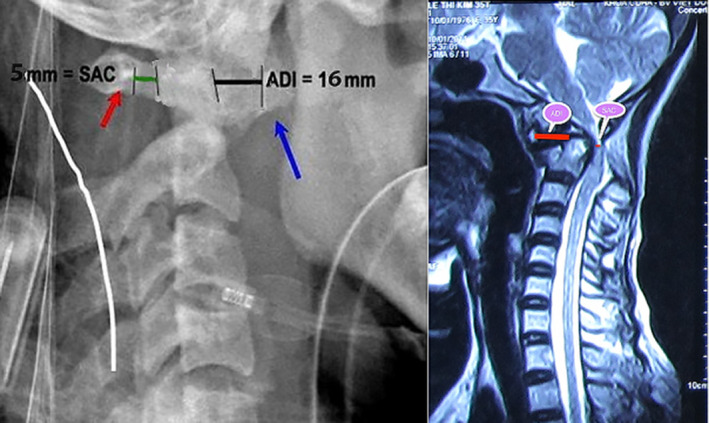
Space available for the spinal cord (SAC). Clinical Case Number 5. Patient A, a 37‐year‐old (before transarticular screw fixation). ADI, American Spinal Injury Association impairment scale.

Orthopaedic and neurological symptoms included pain (VAS score of 7), restricted movement of the cervical spine, impaired motor function (ASIA C grade), and severe neck disability (NDI score of 60%). According to results from radiography, MRI, and CT, the transverse ligament disruption with the anterior displacement of the atlas was greater than 5 mm (type III according to Fielding and Howkins), and spinal cord compression (SAC of 5 mm on MRI (Fig. 6), and an unstable AAD (CAA of 95°) were evident.

The halo device was fixed under local anesthesia and maintained for 2 months. The postoperative CT scan did not confirm full recovery. Type I rotatory displacement (according to Fielding and Howkins) remained. This signaled the formation of a rigid bone‐fibrous block at the site of injury. A complete AAD correction was achieved through the removal of this block with the subsequent stabilization. The patient underwent a transarticular screw fixation with an iliac crest autograft.

**Fig. 7:**
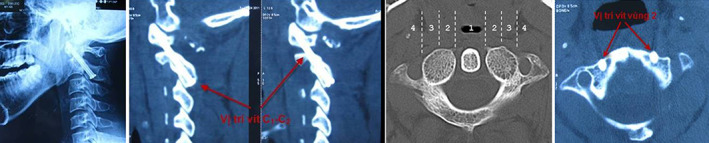
Position of bone graft and C1–C2 fusion 6 months postoperatively. Clinical Case Number 5. Patient A, a 37‐year‐old.

**Fig. 8:**
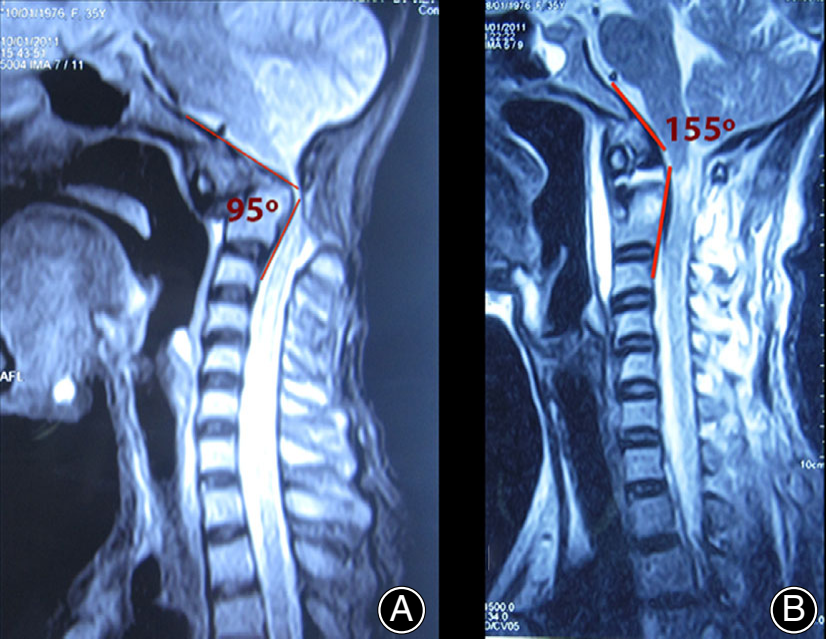
Clivoaxial angle (CAA) before (A) and after (B) transarticular screw fixation. Clinical Case Number 5. Patient A, a 37‐year‐old.

Six months after the operation, complete bone fusion was achieved (Fig. 7) and noticeable clinical improvements were noted: in pain ratings, VAS score of 3; motor function, ASIA D grade; and NDI 40%, moderate disability. The clivoaxial angle was restored to the normal value of 155° (Fig. 8).

### *Clinical Case Number 7*

Patient R, a 31‐year‐old man, was admitted to the SSU on 12 July 2010 with a whiplash injury resulting from a motorcycle accident. Immediately after the accident, he underwent outpatient treatment, but the neck pain persisted. One month after the injury, the patient complained of: pain in the neck and in the shoulder girdle, which tended to become stronger when moving the head and arms; restricted range of neck motion (primarily, bending); headaches in the back part of the head; and dizziness. The patient was kept in a cervical collar until the scheduled surgical treatment.

Orthopaedic and neurological symptoms included pain (VAS score of 6), restricted movement of the cervical spine, impaired motor function (ASIA C grade), and severe neck disability (NDI score of 55%). According to results from radiography, MRI, and CT, the transverse ligament disruption with the anterior displacement of the atlas was greater than 5 mm (type III according to Fielding and Howkins), and spinal cord compression (SAC of 5 mm on MRI), and an unstable AAD (CAA of 97°) were evident.

The halo device was fixed under local anesthesia and maintained for 1.5 months. The postoperative CT scan confirmed full recovery. The patient underwent a transarticular screw fixation with an iliac crest autograft.

Six months after the operation, complete bone fusion was achieved and noticeable clinical improvements were noted: in pain ratings, VAS score of 2; motor function, ASIA D grade; and NDI 35%, moderate disability. The SAC (19 mm) and CAA (154°) returned to normal values.

### *Clinical Case Number 9*

Patient O, a 26‐year‐old woman, was admitted to the SSU on 27 February 2011, after falling during her gymnastics routine. Immediately after the accident, he underwent outpatient treatment, but the neck pain persisted. One and a half months after the injury, the patient complained of: pain in the neck and in the shoulder girdle, which tended to become stronger when moving the head and arms; restricted neck movement; headaches in the back part of the head, radiating to the temple; discomfort in the eye; and blurry vision. The patient was kept in a cervical collar until the scheduled surgical treatment.

Orthopaedic and neurological symptoms included pain (VAS score of 5), restricted movement of the cervical spine, impaired motor function (ASIA D grade), and moderate neck disability (NDI score of 40%). According to results from radiography, MRI, and CT, the transverse ligament disruption with the anterior displacement of the atlas was greater than 5 mm (type III according to Fielding and Howkins), and spinal cord compression (SAC of 5 mm on MRI), and an unstable AAD (CAA of 106°) were evident.

The halo device was fixed under local anesthesia and maintained for 1.5 months. The postoperative CT scan confirmed full recovery. The patient underwent a transarticular screw fixation with an iliac crest autograft.

Six months after the operation, complete bone fusion was achieved and noticeable clinical improvements were noted in pain ratings, with a VAS score of 1. The motor function remained at Grade D. The NDI score improved slightly but remained within the moderate disability range (30%). The SAC score (18 mm) and CAA (150°) returned to normal values.

## Discussion

Thus, halo traction combined with transarticular screw fixation for severe atlantoaxial dislocation allows achievement of postoperative clinical improvement in all patients.

However, posterior transarticular screw fixation carries a potential risk of injury to the vertebral artery, which can be fatal^37^, ^38^. The frequency of vertebral artery injury ranges from 4.1% to 8.2%^39^, ^40^. Examining the isthmus of the axis before surgery using reconstructive CT is recommended. If the isthmus is not wide enough, many surgeons advise against screw fixation^32^, ^33^, ^34^.

Halo reposition allows complete or partial recovery of the spinal cord after compression in acute and severe traumatic AAD and enables reliable fixation of C1–C2 vertebrae. Screw fixation is recommended for most cases of AAD and atlantoaxial instability, as it gives excellent results. Magerl's technique is a common approach for C1–C2 transarticular screw fixation and is preferable in cases of unstable craniocervical injury. In combination with autoplasty, this technique is a biomechanically reliable form of fixation and a first step to bone fusion. However, posterior screw fixation techniques carry a potential risk of vertebral artery injury and require preoperative CT examination of the C2 isthmus.

In this study, halo traction combined with the transarticular screw fixation and bone autoplasty achieved considerable improvements in the neck‐specific measures of functional status, and SAC and CAA measurements.
